# Prevalence and distribution of G6PD deficiency: implication for the use of primaquine in malaria treatment in Ethiopia

**DOI:** 10.1186/s12936-019-2981-x

**Published:** 2019-10-07

**Authors:** Eugenia Lo, Daibin Zhong, Beka Raya, Kareen Pestana, Cristian Koepfli, Ming-Chieh Lee, Delenasaw Yewhalaw, Guiyun Yan

**Affiliations:** 1 Biological Sciences, University of North Carolina at Charlotte, Charlotte, NC 28223 USA; 2 Program in Public Health, College of Health Sciences, University of California, Irvine, CA 92697 USA; 3 School of Medical Laboratory Sciences, Jimma University, Jimma, Ethiopia; 4 Eck Institute for Global Health, University of Notre Dame, Notre Dame, IN 46556 USA; 5 Tropical and Infectious Diseases Research Center, Jimma University, Jimma, Ethiopia

**Keywords:** G6PD deficiency, Malaria, *Plasmodium vivax*, Primaquine, Genotype-phenotype, Ethiopia

## Abstract

**Background:**

G6PD enzyme deficiency is a common enzymatic X-linked disorder. Deficiency of the G6PD enzyme can cause free radical-mediated oxidative damage to red blood cells, leading to premature haemolysis. Treatment of *Plasmodium vivax* malaria with primaquine poses a potential risk of mild to severe acute haemolytic anaemia in G6PD deficient people. In this study, the prevalence and distribution of G6PD mutations were investigated across broad areas of Ethiopia, and tested the association between G6PD genotype and phenotype with the goal to provide additional information relevant to the use of primaquine in malaria treatment.

**Methods:**

This study examined G6PD mutations in exons 3–11 for 344 febrile patient samples collected from seven sites across Ethiopia. In addition, the G6PD enzyme level of 400 febrile patient samples from Southwestern Ethiopia was determined by the CareStart™ biosensor. The association between G6PD phenotype and genotype was examined by Fisher exact test on a subset of 184 samples.

**Results:**

Mutations were observed at three positions of the G6PD gene. The most common G6PD mutation across all sites was A376G, which was detected in 21 of 344 (6.1%) febrile patients. Thirteen of them were homozygous and eight were heterozygous for this mutation. The G267+119C/T mutation was found in 4 (1.2%) individuals in South Ethiopia, but absent in other sites. The G1116A mutation was also found in 4 (1.2%) individuals from East and South Ethiopia. For the 400 samples in the south, 17 (4.25%) were shown to be G6PD-deficient. G6PD enzyme level was not significantly different by age or gender. Among a subset of 202 febrile patients who were diagnosed with malaria, 11 (5.45%) were G6PD-deficient. These 11 infected samples were diagnosed with *Plasmodium vivax* by microscopy. Parasitaemia was not significantly different between the G6PD-deficient and G6PD-normal infections.

**Conclusions:**

The prevalence of G6PD deficiency is modest among febrile patients in Ethiopia. G6PD deficiency testing is thus recommended before administrating primaquine for radical cure of *P. vivax* infected patients. The present study did not indicate a significant association between G6PD gene mutations and enzyme levels.

## Background

Glucose-6-phosphate dehydrogenase (G6PD) is an enzyme involved in the pentose monophosphate pathway. Deficiency of this enzyme leads to free radical-mediated oxidative damage to red blood cells, and in turn causes haemolysis. G6PD deficiency is the most common enzymatic disorder of red blood cells, affecting 400 million people worldwide [1]. It is an X-linked disorder with high prevalence particularly in people of African, Asian, and Mediterranean descent [1]. In Africa, the most common G6PD enzyme-deficient variant is A- [2]. Females with G6PD A- heterozygotes have been shown with selective advantage against severe malaria [3–5]. By selection, this G6PD-deficient trait becomes prevalent (8%) in populations where malaria is endemic [6].

The prevalence of G6PD deficiency is highly relevant to the choice of drug used in anti-malarial treatment [7, 8]. A number of drugs, such as primaquine, dapsone, sulfonamides, quinolones, chloramphenicol, nitrofurantoins (antibiotics), and phenazopyridine (analgesics), have been described as haemolytic trigger that causes haemolytic crisis in G6PD-deficient individuals [9, 10]. Primaquine is the recommended treatment drug to eliminate *Plasmodium vivax* hypnozoites and *Plasmodium falciparum* gametocytes, along with the goal to progress towards zero malaria transmission in Africa [11–14]. It is an ideal agent to be used as primary prophylaxis against *P. vivax* [6]. However, primaquine can also induce oxidative stress causing a spectrum of haemolytic anaemia ranging from mild to severe haemolysis in G6PD-deficient individuals [15]. The likelihood of developing haemolysis and its severity depends on the level of enzyme deficiency, which in turn is determined by the type of G6PD variant [16–18]. The risk for haemolytic anaemia is particularly high in patients who are treated for *P. vivax* malaria because they are usually given a higher dose of primaquine (0.25–0.5 mg in a 14-day treatment regime) compared to those treated for *P. falciparum* (a single dose of 0.25 mg on the first day of treatment) [19]. A high dose of primaquine (0.5 mg base/kg daily for 14 days) has been previously shown to be more effective than a low dose (0.25 mg base/kg daily for 14 days) in eliminating primary blood infection and preventing relapse episodes in *P. vivax* patients [11, 20–22]. However, the lack of G6PD level information, inaccurate methods of screening G6PD deficiency, and the uncertainty in the safety of a single versus long-term primaquine dosage pose risk to malaria patients when treat with primaquine.

The gene encoding the G6PD protein consists of 13 exons and 12 introns [23] and is located on the X chromosome. This gene is highly polymorphic with nearly 160 mutations at the DNA level that are potentially associated with G6PD deficiency [24]. The frequency of these mutations varies among populations and countries. For instance, mutation S188F, sometimes called the Mediterranean mutation, is most prevalent among individuals from the Middle East [25]. Mutations C131G and G487A that were common in Dhaka, Bangladesh appear to be linked to G6PD deficiency by affecting NADP binding or disrupting the protein structure [26]. The G6PD genetic variants were relatively homogeneous in America, Africa, and western Asia compared to those in East Asia and Oceania. In North America, Africa, Yemen and Saudi Arabia, G6PD*A- variant is predominant among populations. By contrast, G6PD variants are highly heterogeneous in East Asia such as China and the Asia–Pacific where no single variant predominates [6, 27].

Ethiopia is one of the few African countries where *P. vivax* and *P. falciparum* coexist, and account for 60% and 40% of the malaria cases, respectively [28]. G6PD deficiency was previously estimated to be as high as 17% in southwestern Ethiopia based on the CareStart™ fluorescence spot test [29]. The mutation A376G that constitutes G6PD*A- variant accounted for nearly 23% of the malaria patients. Other mutations including rs782669677 (535 G->A), rs370658483 (485 + 37 G->T), and chrX:154535443 (C->T) were recently observed in the same geographical region through an investigation of a short segment of the G6PD gene [30]. These mutations did not appear to disrupt function or structure of the G6PD protein [30]. In this study, the prevalence and distribution of G6PD mutations were investigated among a large number of febrile patients across broad areas of Ethiopia. For a subset of samples, the association between G6PD genetic mutations and enzyme level was examined. Malaria parasitaemia and demographic features in the G6PD deficient and non-deficient individuals were characterized.

## Methods

### Study site and sample collection

**Fig. 1:**
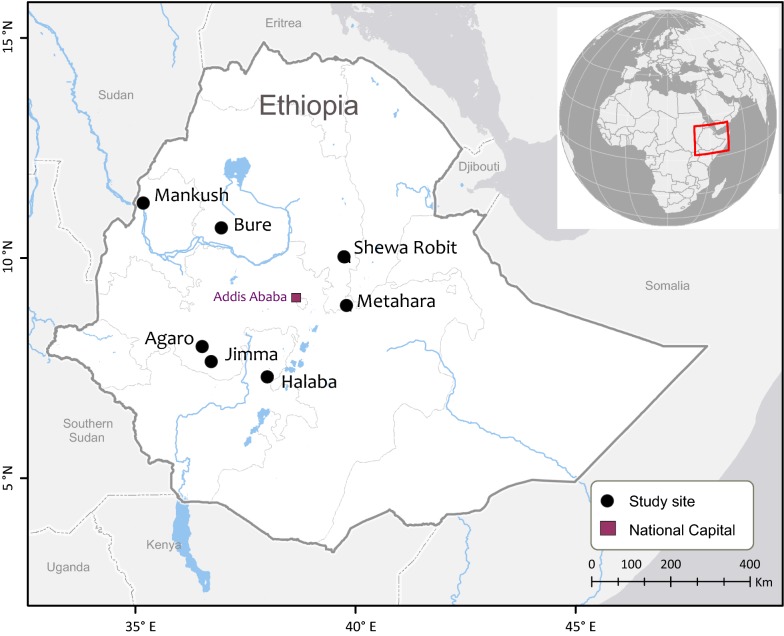
Map showing the distribution of the seven study sites in Ethiopia

A total of 344 blood samples were collected in seven study sites including Bure and Mankush from the north, Metehara and Shewa Robit from the east, as well as Halaba, Agaro, and Jimma from the southwest part of Ethiopia during 2013 to 2016 (Fig. 1). These study sites have high elevations, ranging from 1680 m to 2010 m above sea level. Malaria transmission is seasonal and unstable, with frequent epidemics in these areas. In Bure, Mankush, Metehara, Shewa Robit and Halaba, blood samples from 160 febrile patients visiting the health centers or hospitals were collected and these samples were included in G6PD genotyping. In Agaro and Jimma, blood samples from 184 febrile patients were collected and these samples were included in both G6PD genotyping and phenotyping assays. Genomic DNA was extracted from dried blood spots by the Saponin/Chelex method [31] and eluted in a total volume of 200 μl Tris–EDTA (TE) buffer.

### Molecular genotyping and G6PD gene sequencing

For G6PD genotyping, four PCR assays were conducted to determine the G6PD gene mutations of exon 3–11 (Table 1). For each PCR assay, water was used in a separate reaction as a negative control. PCR amplification was conducted in a 20 μl reaction mixture containing 2 μl of genomic DNA (~ 50 ng/μl), 10 μl of 2 × Maxima SYBR Green qPCR Master Mix (Thermo Fisher) and 0.3 uM of each forward and reverse primers. Amplifications were performed with an initial denaturation at 94 °C for 3 min, followed by 38 cycles at 94 °C for 30 s, 55 °C for 30 s and 72 °C for 60 s, with a final 6 min extension at 72 °C. Amplified PCR products were purified with 2 U Exonuclease I and 2U Shrimp Alkaline Phosphatase (Thermo Fisher) at 37 °C followed by 15 min incubation at 80 °C to deactivate the enzymes. Purified PCR products were sequenced in both directions on an ABI 3730xl DNA analyzer (Genewiz Inc., La Jolla, CA). Sequences were analysed using Codon Code Aligner Program version 7.0.1 (CodonCode Corporation, Centerville, MA). All sequences were aligned to the NCBI reference sequence (NG_009015.2) to verify the specificity of the PCR products. Samples with poor sequencing quality or showed singleton mutations were re-amplified and sequenced.

**Table 1:** PCR primer sequences and amplicon size of G6PD gene

PCR pair	Primer name	Primer sequence (5′–3′)	Primer position	PCR product (bp)	Coding region (bp)	Exon coverage
1	6284F	CAAGGAGTGATTTGGGCAAT	6284			
	6755R	AGAGCAAAACTCCGTCTCCA	6755	472	120	Exon 3
2	D-6F	AATCTCGGGGCTCTTCTGTCTG	16,237			
	D-1170R	GCAACGGCAAGCCTTACATCTG	17,422	1185	365	Exon 4–6
3	D-1880F	CTTCGGGAGGGACCTGCAGAG	18,107			
	D-2865R	GTGGTGACTTCTCCGGGGTTGA	19,114	1007	379	Exon 7–9
4	G6-5F	CCTGAGGGCTGCACATCT	19,356			
	G6-5R	GTGTCTTGCTGATGCCACTG	20,096	740	423	Exon 10–11

Primer position can be referred to NCBI accession no. NG_009015.2

### G6PD phenotype measurement

G6PD enzyme level was measured by CareStart™ Biosensor (Access Bio, Seoul, Korea) on 400 clinical samples collected from Agaro and Jimma, Ethiopia following the manufacturer’s instruction. For each participant, demographic information was recorded by questionnaire. Briefly, for each sample, a G6PD test strip with two drops (20–30 μl) of finger-prick whole blood was inserted into the biosensor at room temperature. The biosensor took about 4–5 min to indicate the G6PD reading and was automatically recorded. The G6PD enzyme activity was expressed in U/dl unit. A blank control was used to calibrate the G6PD biosensor to ensure the reading was zero before the next sample measurement. For each sample that was measured for G6PD enzyme level, haemoglobin level was also estimated using HemoCueHb 201 + analyzer following the manufacturer’s instruction. G6PD enzyme level was normalized by the concentration of haemoglobin (i.e. unit of G6PD enzyme per gram of haemoglobin, U/gHb). G6PD normal and deficient individuals were categorized into two class in male and three classes in female following the WHO classification [11, 32]. The adjusted male median (AMM) G6PD activity, defined as the median G6PD activity of all male participants after excluding samples with less than 10% of the overall median activity, was calculated. For male, class I is G6PD deficient with < 30% of the AMM activity and class II is normal with > 30% of the AMM activity. For female, G6PD activity < 30%, 30–80%, and > 80% of the AMM activity are considered as G6PD deficient, intermediate, and normal, respectively [32].

### Data analysis

All statistical analyses were performed using JMP Pro 12.2.0 software (SAS Institute Inc. 2015). The Mann–Whitney U test was used for non-parametric comparisons, and Student’s t test and one-way analysis of variance for parametric comparisons. Fisher’s exact test was used to examine the association between G6PD genotype and phenotype at the confidence level of *P *= 0.05.

## Results

### Frequency of G6PD gene mutations

A total of 344 febrile patients collected from seven study sites in Ethiopia were sequenced for the G6PD exons 3–11. The common mutation A376G (rs1050829, A→G) was detected in 21 (6.1%) of the individuals, of which ten were homozygote for G376 and eight were heterozygotes (Table 2). This mutation was found in all study sites except Shewa Robit (East Ethiopia). Other mutations including G267+119C/T (rs782000110, C→T) and G1116A (rs2230036, G→A) were detected in 4 (1.2%) and 4 (1.2%) individuals, respectively (Table 2). The G267+119C/T (rs782000110, C→T) mutation was found exclusively in Agaro and Jimma (South Ethiopia); whereas the G1116A (rs2230036, G→A) mutation was found in both East and South Ethiopia. The absence of these mutations in the north could be due to small sample size (*N *= 48; Table 2). All these three mutations were found separately in different individuals. For other previously reported positions [30] such as G202A at exon 4, chrX:154535443 C-T at exon 5, and C563T at exon 6, no mutations were detected among the samples (Table 2). It is noted that the G6PD sequences presented in this study did not cover the two intronic positions (rs370658483, 485 + 37 G→T and rs782669677, 535 G→A) that were previously shown to be polymorphic but rare among the Ethiopians [30].

**Table 2:** Distribution of G6PD genotypes across the seven study sites in Ethiopia

Site	*N*	G6PD genotype											
		A376G		G202A		G267+119C/T		chrX: 154535443 C-T		G1116A		C563T	
		Wild type	Mutant	Wild type	Mutant	Wild type	Mutant	Wild type	Mutant	Wild type	Mutant	Wild type	Mutant
North													
Bure	20	18	2 (G/G)	20	0	20	0	20	0	20	0	20	0
Mankush	28	26	2 (A/G)	28	0	28	0	28	0	28	0	28	0
East													
Metehara	79	73	4 (G/G); 2 (A/G)	79	0	79	0	79	0	77	2 (A/G)	79	0
Shewa Robit	26	26	0	26	0	26	0	26	0	26	0	26	0
South													
Agaro	107	101	4 (G/G); 2 (A/G)	107	0	105	1 (T/T); 1 (C/T)	107	0	106	1 (A/A)	107	0
Halaba	7	4	2 (G/G); 1 (A/G)	7	0	7	0	7	0	7	0	7	0
Jimma	77	75	1 (G/G); 1 (A/G)	77	0	75	1 (T/T); 1 (C/T)	77	0	76	1 (A/A)	77	0
Total	344	323	21 (6.1%)	344	0	340	4 (1.2%)	344	0	340	4 (1.2%)	344	0

Among the 344 febrile patients, a subset of 184 individuals from Agaro and Jimma (South Ethiopia) were analysed further by demography and malaria infection. For the three positions A376G, G267 + 119C-T, and G1116A where mutations were observed, the mutation frequency ranged from 0.9 to 5.1% in males and 2.3 to 5.6% in females. No significant difference was observed in the mutation frequency between males and females (one-tailed *t* test, *P *= 0.47; Table 3). As expected, only hemizygotes (i.e., individuals with only one allele of the G6PD gene) were observed in the male patients, whereas both heterozygotes and homozygote recessive were observed in the female patients.

**Table 3:** Distribution of three G6PD genotypes where mutations were detected among male and female individuals in Jimma and Agaro, southwestern Ethiopia

Gender	*N*	G6PD genotype					
		A376G		G267+119C/T		G1116A	
		Wild type	Mutant	Wild type	Mutant	Wild type	Mutant
Male	118	112 (94.9%)	6 (G/G) (5.1%)	117 (99.1%)	1 (T/T) (0.9%)	116 (98.3%)	2 (A/A) (1.7%)
Female	89	84 (94.4%)	2 (G/G); 3 (A/G) (5.6%)	87 (97.7%)	1 (T/T); 1 (C/T) (2.3%)	89 (100%)	0

Due to the lack of demographic information, samples from other study sites were not included in this analysis

Among the 344 febrile patients, 158 (45.9%) were diagnosed with malaria. Of these 158 malaria patients, the mutation frequency of the three G6PD gene positions (A376G, G267 + 119C-T, and G1116A) ranged from 0.6 to 4.4% (Table 4). Between the malaria-infected and non-infected patients, we found no significant difference in the mutation frequency for A376G, G267 + 119C-T and G1116A (one-tailed t-test, *P *= 0.15; Table 4). Likewise, no significant difference was observed in the mutation frequency when the samples were stratified by age, i.e., under 5, 5–15 and above 14 years old, despite a marked difference in sample size among the three age groups (Additional file 1: Table S1).

**Table 4:** Distribution of G6PD genotypes among non-infected and malaria-infected individuals in Ethiopia

Type of patients	*N*	G6PD genotype					
		A376G		G267+119C/T		G1116A	
		Wild type	Mutant	Wild type	Mutant	Wild type	Mutant
Non-malaria	49	45 (91.8%)	2 (G/G); 2 (A/G) (8.2%)	48 (97.9%)	1 (C/T) (2.1%)	49 (100%)	0
Malaria-infected	158	151 (95.6%)	6 (G/G); 1 (A/G) (4.4%)	157 (99.4%)	2 (T/T) (0.6%)	157 (99.4%)	2 (A/A) (0.6%)

### Measurement of G6PD enzyme activity

**Fig. 2:**
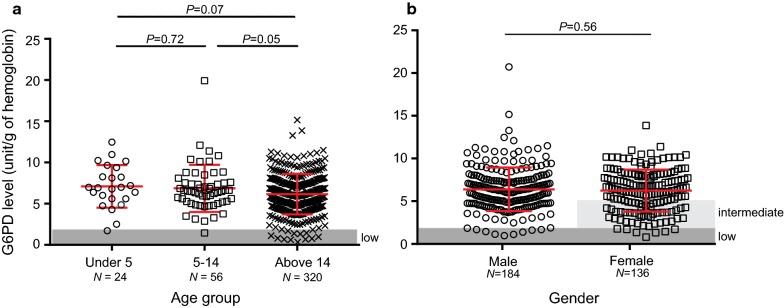
Comparison of G6PD level **a** among three age groups (under 5, 5–14 and above 14 years old) and **b** between males and females. Area in gray indicates samples with G6PD level below 1.88 unit/g of hemoglobin and were considered as deficient. No significant difference was observed in the G6PD level

Based on the WHO guidelines [11, 32], the AMM G6PD activity of all male participants after excluding samples with less than 10% of the overall median activity was 6.25 U/gHb. Thus, patients with value < 1.88 U/gHb (i.e., < 30% of the AMM activity) were considered as G6PD deficient in both male and female. Among the 400 patients from Agaro and Jimma (southwestern Ethiopia), 17 (4.3%) had G6PD activity < 1.88 U/gHb and were considered as G6PD deficient. One of these 17 patients was under the age of 5, one was aged 5–14, and the remaining were above the age of 14 (Fig. 2a). No significant difference was observed in the distribution of G6PD level among the three age groups despite the remarkable difference in sample size (Fig. 2a).

Likewise, no significant difference was observed in the distribution of G6PD enzyme level between males and females (average G6PD level = 6.3 ± 2.5 U/gHb for both gender; one-tailed t-test, *P *= 0.56; Fig. 2b). For male, G6PD level ranged from 0.62 to 20.16 U/gHb, and 10 out of 184 (5.4%) were G6PD deficient. For female, G6PD level ranged from 0.76 to 13.86 U/gHb; 7 out of 136 (5.2%) were G6PD deficient, 47 (34.6%) were G6PD intermediate, and 82 (60.3%) were G6PD normal (Fig. 2b). The prevalence of low G6PD was not significantly different between the two groups.

**Fig. 3:**
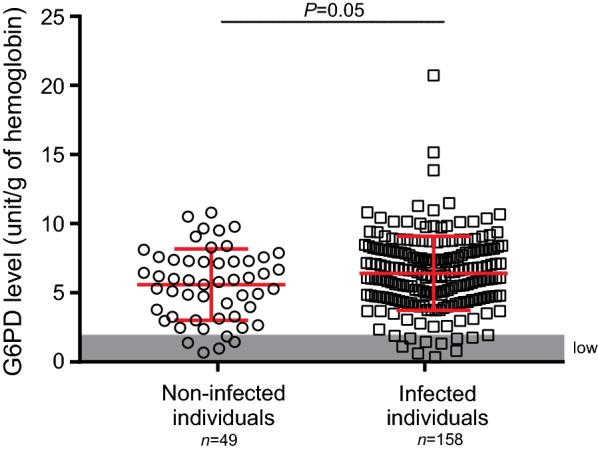
Comparison of G6PD level between non-malaria and malaria-infected individuals. Area in gray indicates samples with G6PD level below 1.88 unit/g of haemoglobin and were considered as G6PD deficient. No significant difference was observed between the two groups

**Fig. 4:**
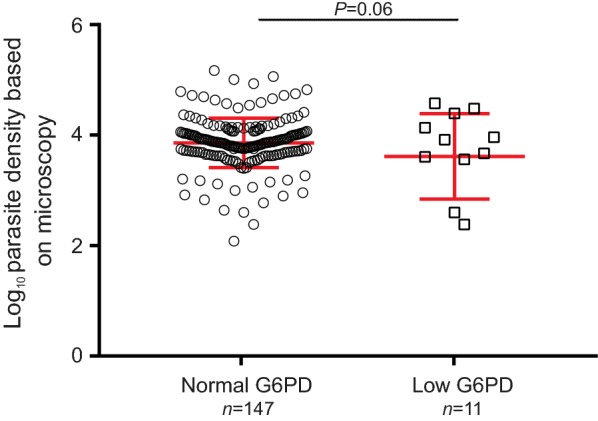
Comparison of microscopic-based parasitaemia between individuals with normal and low G6PD level

Of the 158 patient samples (95 males and 63 females) that were diagnosed with malaria, 11 (6.9%) were considered as G6PD deficient (Fig. 3). For the non-infected individuals, 5 out of 49 (8.2%) were considered as G6PD deficient. Despite the contrast in sample size, there was no significant difference in the distribution of G6PD level between these two groups of individuals (one-tailed t-test, *P *= 0.05; Fig. 3). Among the malaria-infected patients, no significant difference was detected in the microscopic-based parasitaemia between the low and normal G6PD individuals (one-tailed t-test, *P *= 0.06; Fig. 4). Two of the 158 malaria patients were diagnosed with *P. falciparum*, whereas the rest with *P. vivax* infections. The two *P. falciparum*-infected patients had a G6PD level of 4.87 and 5.20 U/gHb, respectively, and were considered as G6PD normal.

### Association between genotype and phenotype

**Fig. 5:**
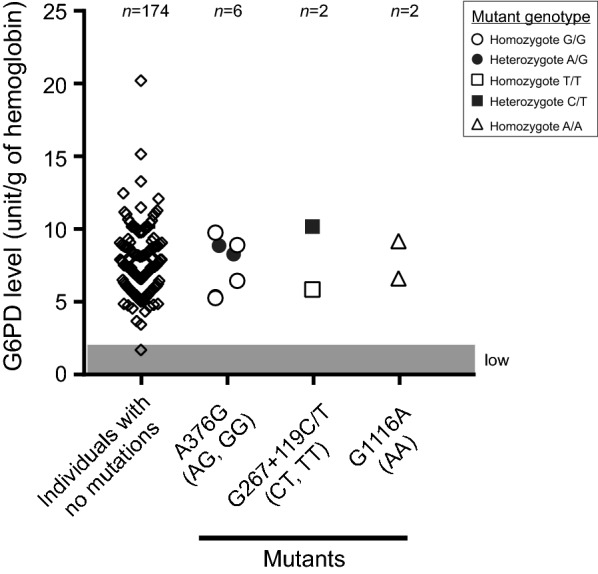
Comparison of G6PD level among individuals without G6PD gene mutations with those with mutations at the different positions including A376G, G267+119C/T and G1116A. Open symbol indicates homozygote and close symbol indicates heterozygote. The corresponding genotypes were indicated below the X-axis. Area in gray indicates samples with G6PD level below 1.88 unit/g of haemoglobin and were considered as G6PD deficient

The G6PD genotype was compared with phenotype for a subset of 184 samples collected from Agaro and Jimma (Table 2). The G6PD enzyme level was compared among the different genotypes (Fig. 5). Of the 174 samples that were indicated with no G6PD mutations, one sample had a G6PD level of 1.69 U/gHb and was considered as G6PD deficient (Fig. 5). For the six samples that had mutation A376G (G/G and A/G), G6PD level ranged from 5.38 to 9.77 U/gHb and all were considered as G6PD normal. For the two samples with mutation G267 + 119C-T (T/T and C/T), G6PD level was normal (5.62 and 10.17 U/gHb, respectively). Likewise, the two samples with mutation G1116A (A/A) also had normal G6PD level (6.61 and 9.17 U/gHb, respectively; Fig. 5). Thus, the mutations observed in A376G, G267 + 119C-T, and G1116A did not associate with low G6PD, despite the limited number of samples in the present study.

## Discussion

To date, 8-aminoquinolines, primaquine and tafenoquine are the only effective drugs that eradicate the dormant liver stages of *P. vivax* and avoid relapse. However, primaquine and tafenoquine can cause acute haemolytic anaemia in individuals with low G6PD enzyme level. The issue of low G6PD has also impacted the use of primaquine as gametocytocide for *P. falciparum* malaria. In the Greater Mekong Subregion, G6PD deficiency has been reported to be common in males with a prevalence of 7.3% to 18.8% based on the CareStart™ Fluorescent Spot Test [33]. In Bangladesh, a prevalence of 17.4% (173/995) for G6PD deficiency (< 60% of the adjusted male median G6PD activity) was reported using standard UV spectrophotometry [34]. Using the CareStart™ G6PD test kit, this study identified a prevalence of 4.3% G6PD deficiency among febrile patients in southwestern Ethiopia based on a threshold cutoff of 1.88 U/g Hb (i.e. < 30% of the AMM G6PD activity). This prevalence rate was slightly higher than a previous report of 1.4% G6PD deficiency in other parts of Ethiopia using the same device and cutoff threshold [35], but much lower than those reported in Asia [33, 34]. Such a pattern indicated differences not only at the continental but also regional level in the distribution of G6PD deficiency.

Some earlier studies demonstrated an association of low G6PD level with a low risk of asymptomatic *P. falciparum* infections [36]; whereas others showed no association of G6PD deficiency with total severe malaria or diseases caused by malaria parasite species [37]. While the potential association between low G6PD level and host immunity is not tested in the present study because of sampling bias towards malaria patients, malaria-infected individuals with normal G6PD enzyme level indicated a slightly but non-significantly higher parasite density than those with low G6PD enzyme. Association analyses did not reveal a significance difference in G6PD enzyme level between males and females, as well as among various age groups. While most of the populations in southwestern Ethiopia belong to either the Amhara or Oromo tribes, ethnicity data of the included patients is not available for us to formally test the association between ethnicity and G6PD enzyme level.

More than 400 allelic variants of the G6PD gene have been reported [10, 18, 26, 38, 39]. Among the 344 Ethiopian samples, three SNPs were detected between exon 3 and exon 11 of the G6PD gene, including codon A376G, one of the most common G6PD mutations with an average frequency of 6.1% across the study sites. This mutation has also been previously reported in in Ethiopia [30, 35, 40]. The other two mutations G267+119C/T and G116A were detected with relatively low frequencies in this study. Based on the analyses of a subset of 184 samples, none of these mutations was associated with low G6PD enzyme level. One possible explanation could be only a small number of low G6PD samples were sequenced. The association between G6PD genotype and phenotype merits further verification with broader samples. Another possibility is that there could be other codons of the G6PD gene that we did not sequence in this study. Also, G6PD phenotype was only measured by one but not multiple biosensors or device for comparison. The CareStart™ G6PD rapid test kit used in this study has the advantages of being small and handy, easy to perform, produce results within a few minutes, and can be used without electricity or specific equipment. It is affordable and can detect G6PD enzyme activity at a very low level [41]. Compared with the gold standard photospectrometric assay, the sensitivity and specificity of the CareStart™ test kit are 90–100% and 84.8–100%, respectively [33, 41, 42], although a low sensitivity was also reported when used on individuals with G6PD enzyme activity < 30% [43, 44]. Though this CareStart™ G6PD test kit represents a significant improvement for quantitative diagnosis of G6PD level over previous models, the accuracy of its measurement still requires further validation before clinical deployment. It is also noteworthy that the samples included in the present study were febrile patients instead of the general population, and that such sampling could bias with a high number of malaria-infected individuals. Thus, this study is limited to inferring the distribution of G6PD level between non-malaria and malaria-infected samples rather than the rate of malaria infection between normal and low G6PD individuals.

## Conclusion

Based on G6PD genotyping, the present study revealed a modest prevalence of G6PD deficiency among febrile patients across Ethiopia. A routine testing for G6PD deficiency prior to administrating primaquine for radical cure of *P. vivax* infected patients is recommend in Ethiopia and other Africa countries. G6PD genotype was not significantly associated with G6PD phenotype. Nevertheless, this association merits further testing with broader samples. Future study should also compare G6PD measurement among different devices with the CareStart™ biosensor to validate accuracy before clinical deployment in Ethiopia as well as other malarious countries.

## Supplementary information

**Additional file 1: Table S1.** Distribution of G6PD genotypes among different age groups. *P *> 0.05 for all pairwise comparison, indicative of no significant difference in mutation frequency among the three age groups.

## Data Availability

All data generated or analysed in this study are included in this published article and its Additional file 1.

## Abbreviations

G6PD: glucose-6-phosphate dehydrogenase
CQ: chloroquine
AMM: adjusted male median
ACT: Artemisinin Combination Therapy

## References

[1] Nkhoma ET, Poole C, Vannappagari V, Hall SA, Beutler E (2009). The global prevalence of glucose-6-phosphate dehydrogenase deficiency: a systematic review and meta-analysis. Blood Cells Mol Dis.

[2] Tishkoff SA, Varkonyi R, Cahinhinan N, Abbes S, Argyropoulos G, Destro-Bisol G (2001). Haplotype diversity and linkage disequilibrium at human G6PD: recent origin of alleles that confer malarial resistance. Science.

[3] Luzzatto L (2012). G6PD deficiency and malaria selection. Heredity (Edinb)..

[4] Sirugo G, Predazzi IM, Bartlett J, Tacconelli A, Walther M, Williams SM (2014). G6PD A- deficiency and severe malaria in The Gambia: heterozygote advantage and possible homozygote disadvantage. Am J Trop Med Hyg.

[5] Manjurano A, Sepulveda N, Nadjm B, Mtove G, Wangai H, Maxwell C (2015). African glucose-6-phosphate dehydrogenase alleles associated with protection from severe malaria in heterozygous females in Tanzania. PLoS Genet.

[6] Howes RE, Piel FB, Patil AP, Nyangiri OA, Gething PW, Dewi M (2012). G6PD deficiency prevalence and estimates of affected populations in malaria endemic countries: a geostatistical model-based map. PLoS Med..

[7] WHO (2015). Guidelines for the treatment of malaria.

[8] Recht J, Ashley EA, White NJ (2018). Use of primaquine and glucose-6-phosphate dehydrogenase deficiency testing: divergent policies and practices in malaria endemic countries. PLoS Negl Trop Dis..

[9] Lee DH, Warkentin TE, Neame PB, Ali MA (1996). Acute hemolytic anemia precipitated by myocardial infarction and pericardial tamponade in G6PD deficiency. Am J Hematol.

[10] Drousiotou A, Touma EH, Andreou N, Loiselet J, Angastiniotis M, Verrelli BC (2004). Molecular characterization of G6PD deficiency in Cyprus. Blood Cells Mol Dis.

[11] WHO. Testing for G6PD deficiency for safe use of primaquine in radical cure of *P. vivax* and *P. ovale* malaria. Geneva: World Health Organization; 2016. https://www.who.int/malaria/publications/atoz/g6pd-testing-pq-radical-cure-vivax/en/.

[12] WHO. Updated WHO Policy Recommendation (October 2012): Single dose Primaquine as a gametocytocide in *Plasmodium falciparum* malaria. Geneva: World Health Organization; 2012. https://www.who.int/malaria/publications/atoz/who_pq_policy_recommendation/en/.

[13] Domingo GJ, Satyagraha AW, Anvikar A, Baird K, Bancone G, Bansil P (2013). G6PD testing in support of treatment and elimination of malaria: recommendations for evaluation of G6PD tests. Malar J..

[14] Fernando D, Rodrigo C, Rajapakse S (2011). Primaquine in vivax malaria: an update and review on management issues. Malar J..

[15] Pamba A, Richardson ND, Carter N, Duparc S, Premji Z, Tiono A (2012). Clinical spectrum and severity of hemolytic anemia in glucose 6-phosphate dehydrogenase–deficient children receiving dapsone. Blood.

[16] Mason PJ, Bautista JM, Gilsanz F (2007). G6PD deficiency: the genotype-phenotype association. Blood Rev.

[17] Cappellini MD, Fiorelli G (2008). Glucose-6-phosphate dehydrogenase deficiency. Lancet.

[18] Nantakomol D, Paul R, Palasuwan A, Day NP, White NJ, Imwong M (2013). Evaluation of the phenotypic test and genetic analysis in the detection of the glucose-6-phosphate dehydrogenase deficiency. Malar J..

[19] WHO. Control and elimination of *Plasmodium vivax* malaria—a technical brief. Geneva: World Health Organization; 2015. https://www.who.int/malaria/publications/atoz/9789241509244/en/.

[20] Hill DR, Baird JK, Parise ME, Lewis LS, Ryan ET, Magill AJ (2006). Primaquine: report from CDC expert meeting on malaria chemoprophylaxis. Am J Trop Med Hyg.

[21] Gonzalez-Ceron L, Rodriguez MH, Sandoval MA, Santillan F, Galindo-Virgen S, Betanzos AF (2015). Effectiveness of combined chloroquine and primaquine treatment in 14 days versus intermittent single dose regimen, in an open, non-randomized, clinical trial, to eliminate *Plasmodium vivax* in southern Mexico. Malar J..

[22] Durand S, Cabezas C, Lescano AG, Galvez M, Gutierrez S, Arrospide N (2014). Efficacy of three different regimens of primaquine for the prevention of relapses of *Plasmodium vivax* malaria in the Amazon Basin of Peru. Am J Trop Med Hyg.

[23] Martini G, Toniolo D, Vulliamy T, Luzzatto L, Dono R, Viglietto G (1986). Structural analysis of the X-linked gene encoding human glucose 6-phosphate dehydrogenase. EMBO J.

[24] Gomez-Manzo S, Terron-Hernandez J, de la Mora I, Garcia-Torres I, Lopez-Velazquez G, Reyes-Vivas H (2013). Cloning, expression, purification and characterization of his-tagged human glucose-6-phosphate dehydrogenase: a simplified method for protein yield. Protein J..

[25] Doss CG, Alasmar DR, Bux DI, Sneha P, Bakhsh FD, Al-Azwani I (2016). Genetic epidemiology of glucose-6-phosphate dehydrogenase deficiency in the Arab World. Sci Rep..

[26] Sarker SK, Islam MT, Eckhoff G, Hossain MA, Qadri SK, Muraduzzaman AK (2016). Molecular analysis of glucose-6-phosphate dehydrogenase gene mutations in Bangladeshi Individuals. PLoS One.

[27] Beutler E (1994). G6PD deficiency. Blood.

[28] Lo E, Yewhalaw D, Zhong D, Zemene E, Degefa T, Tushune K (2015). Molecular epidemiology of *Plasmodium vivax* and *Plasmodium falciparum* malaria among Duffy-positive and Duffy-negative populations in Ethiopia. Malar J..

[29] Tsegaye A, Golassa L, Mamo H, Erko B (2014). Glucose-6-phosphate dehydrogenase deficiency among malaria suspects attending Gambella hospital, southwest Ethiopia. Malar J..

[30] Carter TE, Mekonnen SK, Lopez K, Bonnell V, Damodaran L, Aseffa A (2018). Glucose-6-phosphate dehydrogenase deficiency genetic variants in malaria patients in Southwestern Ethiopia. Am J Trop Med Hyg.

[31] Bereczky S, Martensson A, Gil JP, Farnert A (2005). Rapid DNA extraction from archive blood spots on filter paper for genotyping of *Plasmodium falciparum*. Am J Trop Med Hyg.

[32] WHO. In vitro diagnostics medical devices to identify Glucose-6-phosphate dehydrogenase (‎G6PD)‎ activity. Geneva: World Health Organization; 2016. https://apps.who.int/iris/handle/10665/252628. Accessed 25 Apr 2019.

[33] Bancone G, Menard D, Khim N, Kim S, Canier L (2019). Molecular characterization and mapping of glucose-6-phosphate dehydrogenase (G6PD) mutations in the Greater Mekong Subregion. Malar J..

[34] Ley B, Alam MS, O’Donnell JJ, Hossain MS, Kibria MG, Jahan N (2017). A comparison of three quantitative methods to estimate G6PD activity in the Chittagong Hill Tracts, Bangladesh. PLoS One..

[35] Shitaye G, Gadisa E, Grignard L, Shumie G, Chali W, Menberu T (2018). Low and heterogeneous prevalence of glucose-6-phosphate dehydrogenase deficiency in different settings in Ethiopia using phenotyping and genotyping approaches. Malar J..

[36] Amoah LE, Opong A, Ayanful-Torgby R, Abankwa J, Acquah FK (2016). Prevalence of G6PD deficiency and *Plasmodium falciparum* parasites in asymptomatic school children living in southern Ghana. Malar J..

[37] Mbanefo EC, Ahmed AM, Titouna A, Elmaraezy A, Trang NT, Phuoc Long N (2017). Association of glucose-6-phosphate dehydrogenase deficiency and malaria: a systematic review and meta-analysis. Sci Rep..

[38] Louicharoen C, Nuchprayoon I (2005). G6PD Viangchan (871G > A) is the most common G6PD-deficient variant in the Cambodian population. J Hum Genet.

[39] Chen Y, Xiu W, Dong Y, Wang J, Zhao H, Su Y (2018). Mutation of glucose-6-phosphate dehydrogenase deficiency in Chinese Han children in eastern Fujian. Medicine (Baltimore)..

[40] Assefa A, Ali A, Deressa W, Tsegaye W, Abebe G, Sime H (2018). Glucose-6-phosphate dehydrogenase (G6PD) deficiency in Ethiopia: absence of common African and Mediterranean allelic variants in a nationwide study. Malar J..

[41] von Fricken ME, Weppelmann TA, Eaton WT, Masse R, Rochars MV, Okech BA (2014). Performance of the CareStart™ glucose-6-phosphate dehydrogenase (G6PD) rapid diagnostic test in Gressier, Haiti. Am J Trop Med Hyg..

[42] Adu-Gyasi D, Asante KP, Newton S, Dosoo D, Amoako S, Adjei G (2015). Evaluation of the diagnostic accuracy of CareStart™ G6PD deficiency Rapid Diagnostic Test (RDT) in a malaria endemic area in Ghana, Africa. PLoS One..

[43] Kim S, Nguon C, Guillard B, Duong S, Chy S, Sum S (2011). Performance of the CareStart™ G6PD deficiency screening test, a point-of-care diagnostic for primaquine therapy screening. PLoS One.

[44] Monteiro WM, Brito MAM, Lacerda MVG (2017). Accuracy of CareStart™ G6PD rapid diagnostic test: variation in results from different commercial versions. Rev Soc Bras Med Trop.

