# Supplementary material for: Prevalence and distribution of G6PD deficiency: implication for the use of primaquine in malaria treatment in Ethiopia
Source: Malar J. 2019 Oct 7;18:340. doi: 10.1186/s12936-019-2981-x (PMC6781416; doi:10.1186/s12936-019-2981-x)
Supplement: Supplementary file 1 — Additional file 1: Table S1. Distribution of G6PD genotypes among different age groups. P > 0.05 for all pairwise comparison, indicative of no significant difference in mutation frequency among the three age groups. [file 12936_2019_2981_MOESM1_ESM.docx]

Additional file 1: Table S1. Distribution of G6PD genotypes among different age groups. *P* > 0.05 for all pairwise comparison, indicative of no significant difference in mutation frequency among the three age groups.

| **Age group** | ***N*** | **G6PD genotype** | | | | | | | | | | | |
| --- | --- | --- | --- | --- | --- | --- | --- | --- | --- | --- | --- | --- | --- |
|  |  | **A376G** | | **G202A** | | **G267+119C/T** | | **chrX: 154535443 C-T** | | **G1116A** | | **C563T** | |
|  |  | **Wild type** | **Mutant** | **Wild type** | **Mutant** | **Wild type** | **Mutant** | **Wild type** | **Mutant** | **Wild type** | **Mutant** | **Wild type** | **Mutant** |
| **Under 5** |  |  |  |  |  |  |  |  |  |  |  |  |  |
|  | 17 | 17  (100%) | 0 | 17  (100%) | 0 | 17  (100%) | 0 | 17  (100%) | 0 | 17  (100%) | 0 | 17  (100%) | 0 |
| **5 - 14** |  |  |  |  |  |  |  |  |  |  |  |  |  |
|  | 44 | 42  (95.5%) | 2 (G/G) (4.5%) | 44  (100%) | 0 | 42  (95.5%) | 2(T/T)  (4.5%) | 44  (100%) | 0 | 44  (100%) | 0 | 44  (100%) | 0 |
| **Above 14** | |  |  |  |  |  |  |  |  |  |  |  |  |
|  | 146 | 137  (93.8%) | 6 (G/G);  3(A/G)  (6.2%) | 158  (100%) | 0 | 145  (99.3%) | 1(C/T)  (0.7%) | 158  (100%) | 0 | 144  (98.6%) | 2 (A/A)  (1.4%) | 158  (100%) | 0 |
